# Human Cardiac Progenitor Spheroids Exhibit Enhanced Engraftment Potential

**DOI:** 10.1371/journal.pone.0137999

**Published:** 2015-09-16

**Authors:** Francesca Oltolina, Andrea Zamperone, Donato Colangelo, Luca Gregoletto, Simone Reano, Stefano Pietronave, Simone Merlin, Maria Talmon, Eugenio Novelli, Marco Diena, Carmine Nicoletti, Antonio Musarò, Nicoletta Filigheddu, Antonia Follenzi, Maria Prat

**Affiliations:** 1 Dept. Health Sciences, Università del Piemonte Orientale “A. Avogadro”, Novara, Italy; 2 Dept. Translational Medicine, Università del Piemonte Orientale “A. Avogadro”, Novara, Italy; 3 Dept. of Cardiac Surgery, Clinica S. Gaudenzio, Novara, Italy; 4 Institute Pasteur Cenci-Bolognetti, DAHFMO, Roma, Italy; 5 Unit of Histology and Medical Embryology, IIM, Sapienza University of Rome, Rome, Italy; 6 Centro di Biotecnologie per la Ricerca Medica Applicata (BRMA), Novara, Italy; University-Hospital of Parma, ITALY

## Abstract

A major obstacle to an effective myocardium stem cell therapy has always been the delivery and survival of implanted stem cells in the heart. Better engraftment can be achieved if cells are administered as cell aggregates, which maintain their extra-cellular matrix (ECM). We have generated spheroid aggregates in less than 24 h by seeding human cardiac progenitor cells (hCPCs) onto methylcellulose hydrogel-coated microwells. Cells within spheroids maintained the expression of stemness/mesenchymal and ECM markers, growth factors and their cognate receptors, cardiac commitment factors, and metalloproteases, as detected by immunofluorescence, q-RT-PCR and immunoarray, and expressed a higher, but regulated, telomerase activity. Compared to cells in monolayers, 3D spheroids secreted also bFGF and showed MMP2 activity. When spheroids were seeded on culture plates, the cells quickly migrated, displaying an increased wound healing ability with or without pharmacological modulation, and reached confluence at a higher rate than cells from conventional monolayers. When spheroids were injected in the heart wall of healthy mice, some cells migrated from the spheroids, engrafted, and remained detectable for at least 1 week after transplantation, while, when the same amount of cells was injected as suspension, no cells were detectable three days after injection. Cells from spheroids displayed the same engraftment capability when they were injected in cardiotoxin-injured myocardium. Our study shows that spherical *in vivo* ready-to-implant scaffold-less aggregates of hCPCs able to engraft also in the hostile environment of an injured myocardium can be produced with an economic, easy and fast protocol.

## Introduction

The demonstration that all tissues in the organism contain stem cells has opened to the new possibility of cell therapy and regenerative medicine in case of organ injury [1]. Stem cell transplantation has proven to be a promising strategy for the treatment of ischemic cardiovascular diseases [2], which are the leading cause of mortality and morbidity worldwide and have high socioeconomic costs [3,4]. The recently developed cell therapies, aimed at replacing the injured lost myocardial cells, may provide new opportunities to treat cardiac infarct, and indeed clinical trials have already started, although so far with modest results [5,6].

When applying cell therapy to an injured organ, a crucial point is the conformation to the properties of the damaged tissue to be repaired or replaced. Thus, the cell type and the way or the form for their delivery have a pivotal role. In the case of myocardium, among the various cell types that have been proposed as candidates the cardiac progenitor cells (CPC) seems to be the most promising [2]. In fact, other cell sources, like skeletal muscle satellite cells, bone marrow derived mesenchymal stem cells, adipose tissue derived mesenchymal stromal cells, amniotic fluid derived cells, do not properly integrate within the myocardium [2].

The potential of CPCs is likely related to the fact they are already committed to their destiny [2], having received the influence of the cardiac environment, and thus are more prone to differentiate towards the required phenotype. Indeed they are responsible for the myocardial homeostasis throughout lifetime [7]. CPCs still retain their multipotency, being able to give origin also to endothelial and smooth muscle cells, besides cardiomyogenic cells [8].

Human CPCs (hCPCs) are generally identified for the expression of biochemical markers, such as c-kit, MDR, Sca-1, NKX2.5, CD105 [8–10], whose expression, however, is not restricted to this cell population and in some cases was found to be unstable [9,11]. For this reason, the identifying criteria for hCPCs are still debated. hCPCs can be identified also on the basis of functional properties, such as the ability to form cardiospheres [12]. Notwithstanding these uncertainties, clinical trials with hCPCs are already under way [5,6,13].

As already pointed out, the form and the method of *in vivo* delivery plays a key role for a successful engraftment. Indeed, since the first *in vivo* cell injection experiments and treatments for cardiac repair it has been evident that most cells are lost in the first 24 hours, and that their engraftment was always inadequate [14]. To overcome these limitations, therapies were pursued by *ex vivo* cardiac tissue engineering to produce 3D structures containing the cellular component supported by a biomimetic scaffold [15–17]. The possibility to produce scaffold-less multicellular aggregates, such as cardiospheres, which are obtained by expanding clonal derived cells as self-adherent clusters in suspension [12], or purposely produced cell sheets [18–20] and sheet fragments [21], from which cells migrate and establish contacts with the resident cells in the myocardium, opened new possibilities since, in principle, the inflammatory reaction triggered by the scaffold should be avoided. Moreover, the extracellular matrix produced by these cells is not lost as a consequence of the enzymatic processing necessary for the recovery of the cells to be transplanted as cell suspension; on the contrary, it should favour their adhesion to the myocardium and survival, although still in a low number [22]. These observations have suggested a beneficial effect most probably due to a paracrine indirect mechanism, rather than a direct structural involvement [23]. Both cardiospheres and cell sheets require relatively long-term cultures and expensive reagents. A cell culture system employing methylcellulose (MC) hydrogel wells was reported to allow the production of implantable spherical clusters made of rat bone marrow mesenchymal stem cells [24–26] and human amniotic cells [27].

The advantage of this method is that spherical symmetric spheres can be obtained in less than 24 h, with low cost reagents and with an easy approach. We have thus decided to investigate whether such a strategy could apply also to hCPCs. Cells within spheroids were found to maintain the properties of the original cells cultured as 2D monolayers and, in the meantime, to display a higher migration potential and a better in vivo engraftment, both in healthy and in cardiotoxin-injured myocardium.

## Methods

### Isolation of hCPCs and preparation of spheroids

**Fig 1:**
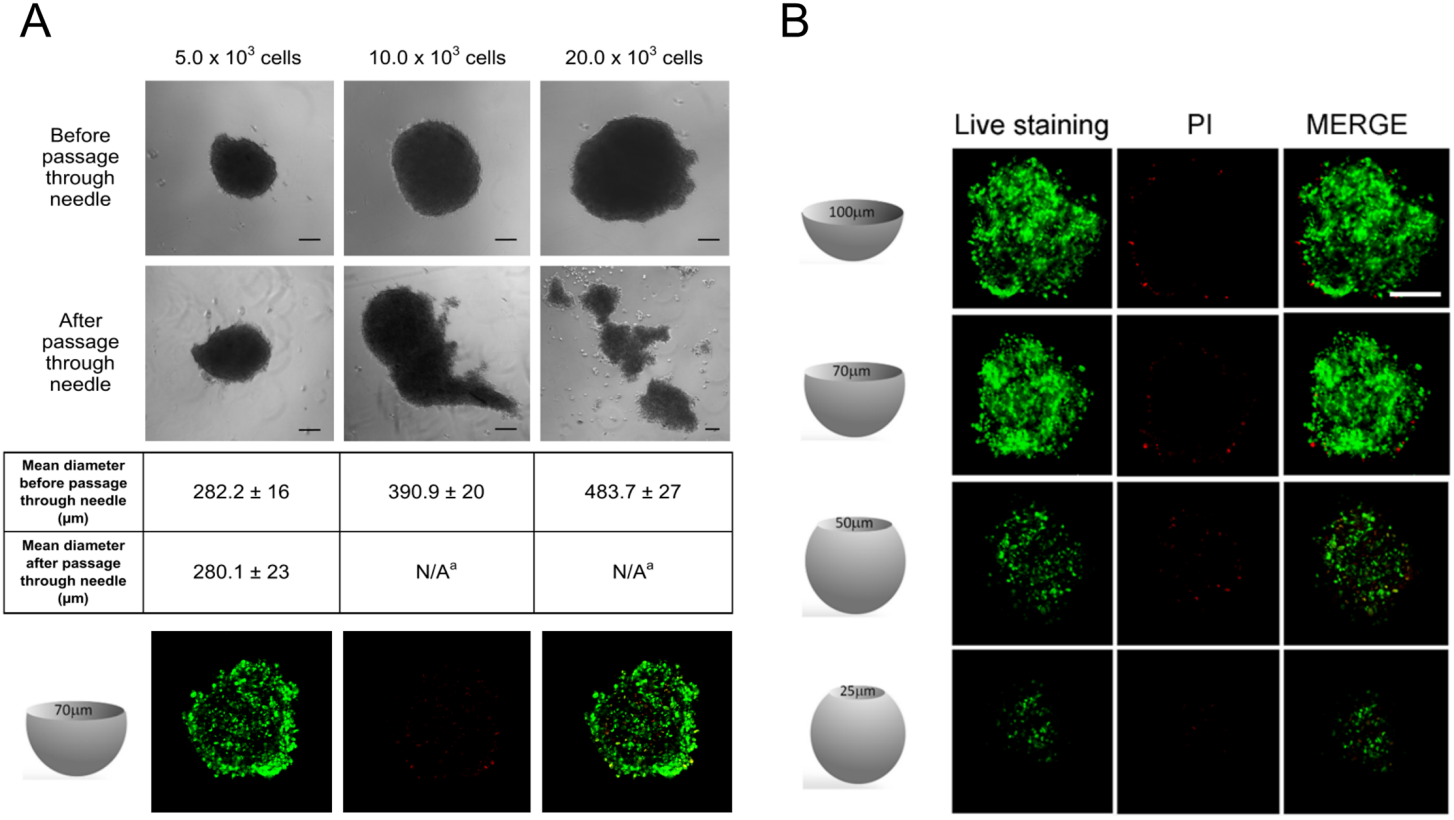
Morphology and viability of hCPC spheroids. (**A**) hCPC spheroids obtained from 5x10^3^, 10x10^3^ or 25x10^3^ cells before and after their passage through a 27G needle (scale bar, 100 μm). (**B**) Viability of spheroids as evaluated in a Live/Dead assay (viable cells in green, PI-positive dead cells in red). Scale bar, 100 μm. Four optical sections of the spheroids taken at different levels of the z-axis are reported. Live/Dead assay was performed also on 5x10^3^ cells spheroids after passage through the needle (A, bottom). One representative out of the three experiments performed with similar results is shown.

hCPCs were isolated from samples of right auricula of patients (N = 8, age range: 50–86; median age: 74.5±11.8; 5 males and 3 females), undergoing cardiac surgery at the Department of Cardiac Surgery of the Clinic of St. Gaudenzio, Novara (Italy). Signed written informed consents were obtained according to a protocol approved by the Institutional Review Board (IRB) of Novara (Italy). (Comitato Etico Interaziendale, protocol #338/CE, study CARDIOCELL, CE 54/10, approved on June 22, 2010). Auricula fragments were processed as already described [11,19]. Tissue fragments were plated on 0.02% gelatin-coated dishes in 1/3 Claycomb (Sigma Aldrich, St. Louis, MO, USA) and 2/3 F12K (Invitrogen Life Technologies Italia, Monza MB, Italy), 10% fetal bovine serum (FBS) (Lonza Biowittaker, Verviers, Belgium), 100 IU/ml penicillin and 100 μg/ml streptomycin (Sigma Aldrich), and cultured at 37°C in 5% CO_2_. Cells migrating from the fragments were sorted by magnetic immunobeads using anti-c-kit antibody (Miltenyi Biotec GmbH, Germany). These cells were positive for c-kit, CD90, Sca-1, CD44, CD105, and negative for CD34 and CD45 as previously reported, although c-kit expression was lost after few passages [11,19]. Spheroids were prepared with cells grown for 5 to 7 passages [11]. hCPCs were dissociated from culture dishes by trypsinization, then seeded drop-wise in the multiwell methylcellulose and cultured for 24 h (12% MC, aqueous solution wt/vol) under standard conditions. Hydrogel system was prepared as described [26,27]. Spheroids were prepared using different amount of cells, ranging from 5x10^3^ to 25x10^3^. For this work, most of the spheroids were produced with an average of 5x10^3^. These spheroids had a size compatible for the *in vivo* studies, since they were able to pass throughout a 27 gauge-needle maintaining full cell viability and cells did not dissociate (Fig 1A).

### Characterization and properties of hCPC spheroids

#### a) Morphological analysis

hCPC spheroids were analysed using a Leica DMRB microscope equipped with a digital camera. Photomicrographs were taken and spheroid diameters were measured by using a computer-based image analysis system (ImageJ, NIH, USA).

#### b) Cell viability

hCPCs viability within spheroids was evaluated with the Live-Dead Cell Staining Kit (Biovision, Milpitas, California, USA). Freshly stained cells were analysed by laser scanning confocal microscope (Leica TCS SP2, Heerbrugg, Switzerland). Live-Dead staining was performed also after the passage of spheroids through a 27-gauge needle syringe, in order to control cell viability, besides spheroid shape and size.

#### c) Immunofluorescence

hCPC spheroids were washed with phosphate buffer saline (PBS), fixed with 3% paraformaldehyde (PFA) for 20 min, rinsed with PBS, and permeabilised with 0.2% Triton X-100, 1% bovine serum albumin (BSA), 5% fetal bovine serum (FBS) in phosphate buffered saline (PBS) for 2 h. Cells in monolayers and spheroids were incubated for 1 h with primary antibodies (listed in Table 1) dissolved in PBS-0.2% Triton X-100, 1% BSA, then washed three times with PBS-0.2% Triton X-100, and incubated with the appropriate FITC-conjugated (Abcam, Cambridge, MA, USA, excitation 488 nm and emission 520 nm) secondary antibodies. Nuclei were stained with TO-PRO-3® (1/100, Life Technologies Italy, excitation at 642 nm and emission at 661 nm), cytoskeletal actin was stained using Alexa Fluor 546-phalloidin ((1/200, Life Technologies Italy, excitation 556 nm and emission at 570 nm). Fluorescence was detected using a Leica TCS SP2 AOBS spectral confocal scanner microscope. ImageJ software was used for image analysis.

**Table 1:** Primary antibodies used for immunofluorescence.

Antigen	Species	Dilution	Source	Cat. No
Fibronectin	Rabbit polyclonal	1/200	Sigma-Aldrich	F3648
Connexin 43	Rabbit polyclonal	1/200	AbCam	Ab11370
Collagen I	Rabbit polyclonal	1/500	AbCam	Ab34710
Vimentin	Mouse monoclonal	1/40	Sigma-Aldrich	V6630
Laminin	Rabbit polyclonal	1/50	Dako	Z0097
GATA-4	Rabbit polyclonal	1/100	AbCam	Ab61170
MEF 2C	Rabbit polyclonal	1/100	AbCam	Ab64644
CD44	Rat monoclonal	1/10	BioLegend	103021
CD90	Mouse monoclonal	1/10	BioLegend	328108
CD117/c-kit	Mouse monoclonal	1/10	BioLegend	323404
Yap	Rabbit polyclonal	1/100	Santa Cruz	15407
Met	Mouse monoclonal	1/100	Property	Prat, 1991)
Sca-1	Mouse monoclonal	1/50	Ebioscience	14-5981-85
Human nuclear antigen	Mouse monoclonal	1/100	Millipore	mAb 1281

#### d) Quantitative real-time PCR (qPCR)

Total RNA was extracted in Trizol® (Invitrogen Life Technologies Italy), followed by DNAse treatment (DNAse I) (Fermentas, St. Leon-Rot, Germany). Then, 1 μg RNA was retrotranscribed in cDNA with the RevertAid™ H Minus First Strand cDNA Synthesis Kit (Fermentas) using the oligo(dT) primers. Each gene expression was normalized on the housekeeping gene ribosomal 18S rRNA. Analysis were based on the methods previously described and adapted in our laboratory [11]. Briefly, assays were performed in triplicate for each treatment in a 20 μl reaction volume containing 1 μl of RT products, 10 μl Sso-Fast EVA Green SMX (Bio-Rad), 500 nM each forward and reverse primers, as indicated in Table 2. An automated CFX96 real-time thermocycler was used (Bio-Rad). The reaction conditions were 95°C for 1 minute, followed by 45 cycles 98°C for 5 seconds and anneal–extend step for 5 seconds at 60°C (Table 2), with data collection. At the end of these cycles, a melting curve (65°C to 95°C, with plate read every 0.5°C) was performed in order to assess the specificity of the amplification product by single peak melting temperature verification (e.g. 18S rRNA melting temperature peak was 86°C). Results were analysed with Bio-Rad CFX Manager and exported to Excel (Microsoft, Redmond, WA, USA) for calculation and statistical analyses.

**Table 2:** Primers used for qPCR.

Gene		Sequence (5’– 3’)
18S rRNA	Fw	GTGGAGCGATTTGTCTGGTT
	Rv	ACGCTGAGCCAGTCAGTGTA
β-ACTIN	Fw	ACTTCGAGCAAGAGATGGCC
	Rv	CACATCTGCTGGAAGGTGG
β-GLOBIN	Fw	CGGCGGCGGGCGGCGCGGGCTGGGCGGCTTCATCCACGTTCACCTTG
	Rv	GCCCGGCCCGCCGCGCCCGTCCCGCCGGAGGAGAAGTCTGCCGTT
c-MET	Fw	GGGTCGCTTCATGCAGGTTGTGGT
	Rv	ATGGTCAGCCTTGTCCCTCCTTCA
COLLAGEN I	Fw	AAGGTCATGCTGGTCTTGCT
	Rv	GACCCTGTTCACCTTTTCCA
COLLAGEN IV	Fw	ACTCTTTTGTGATGCACACCA
	Rv	AAGCTGTAAGCGTTTGCGTA
FN1	Fw	AGACCCCAGGCACCTATCAC
	Rv	TCGGTCACTTCCACAAACTG
GATA-4	Fw	ACCTGGGACTTGGAGGATAGCAAA
	Rv	TCCCATCAGCGTGTAAAGGCATCT
HGF	Fw	ACGCTACGAAGTCTGTGACA
	Rv	CAAGAGTATAGCACCATGGC
HMBS	Fw	GGCAATGCGGCTGCAA
	Rv	GGGTACCCACGCGAATCAC
hTERT	Fw	GACGTGGAAGATGAGCGTG
	Rv	GACGACGTACACACTCATC
IGF-1	Fw	CTTCAGTTCGTGTGTGGAGACAG
	Rv	CGCCCTCCGACTGCTG
IGF-1R	Fw	CTCCTGTTTCTCTCCGCCG
	Rv	ATAGTCGTTGCGGATGTCGAT
IGF-2R	Fw	GAGGGAAGAGGCAGGAAAG
	Rv	TGTGGCAGGCATACTCAG
ITGA1	Fw	GGTTCCTACTTTGGCAGTATT
	Rv	AACCTTGTCTGATTGAGAGCA
ITGB4	Fw	AGACGAGATGTTCAGGGACC
	Rv	GGTCTCCTCTGTGATTTGGAA
MEF-2C	Fw	GACTGTGAGATTGCGCTGAT
	Rv	CGTCTCCACGATGTCTGAGT
MEF-2D	Fw	CCCCTGCTGGAGGACAAGTA
	Rv	CCCCTGCTGGAGGACAAGTA
MMP-2	Fw	GGCCCTGTCACTCCTGAGAT
	Rv	GGCATCCAGGTTATCGGGGA
MMP-14	Fw	CCCTATGCCTACATCCGTGA
	Rv	TCCATCCATCACTTGGTTAT
NKX2.5	Fw	CGCCGCTCCAGTTCATAG
	Rv	GGTGGAGCTGGAGAAGACAGA
SDF-1	Fw	GGTGGAGCTGGAGAAGACAGA
	Rv	CAGCCGGGCTACAATCTGAA
CXCR4	Fw	TGACGGACAAGTACAGGCTGC
	Rv	CCAGAAGGGAAGCGTGATGA
Telomere	Fw	ACACTAAGGTTTGGGTTTGGGTTTGGGTTTGGGTTAGTGT
	Rv	TGTTAGGTATCCCTATCCCTATCCCTATCCCTATCCCTAACA
VE-cad 2	Fw	GGCATCTTCGGGTTGATCCT
	Rv	CCGACAGTTGTAGGCCCTGTT
VEGF	Fw	TGCAGATTATGCGGATCAAACC
	Rv	TGCATTCACATTTGTTGTGCTGTAG
VEGFR-1	Fw	CAGGCCCAGTTTCTGCCATT
	Rv	TTCCAGCTCAGCGTGGTCGTA
VEGFR-2	Fw	CCAGCAAAAGCAGGGAGTCTGT
	Rv	TGTCTGTGTCATCGGAGTGATATCC
VLA-4	Fw	ATCTGACTCTGCCTTCAT
	Rv	CATTCCTCACCATCACTG

#### e) Growth Factors and receptors protein array

Cell culture media were collected from hCPCs monolayers and spheroids kept in serum free medium for two days. Extracts were prepared by lysing cells with iced RIPA buffer (20mM Tris-HCl pH 7.5, 150mM NaCl, 50mM HEPES, 0.1% SDS, 1mM EGTA, 1% NP-40, 1% sodium deoxycholate, 2.5mM sodium pyrophosphate, 10% glycerol, and a cocktail of protease inhibitors (Sigma- Aldrich)). Protein content was quantified by Bicinchoninic acid (BCA; Sigma-Aldrich) assay. Both culture supernantants and cell extracts were analysed for their content of growth factors and receptors by incubation on membranes of the RayBio® C-Series Human Growth Factor Antibody Array C1 kit (RayBiotech, Norcross, GA, USA), following manufacturer’s instructions (see Table 3 for array map). Briefly, after incubation with blocking buffer, undiluited culture media or 140 μg of proteins extracted from cells were incubated on the kit membranes overnight at 4°C. Several washes were performed and the biotinylated antibody cocktail was added for 2 hours at room temperature followed by a HRP-streptavidin incubation. A chemiluminescence imaging system was used to collect the data and analysis were performed by using a computer-based image analysis system (ImageJ, NIH, USA).

**Table 3:** Antibody array scheme.

	A	B	C	D	E	F	G	H	I	J	K	L
1	POS	POS	NEG	NEG	AREG	bFGF	b-NGF	EGF	EGF R	FGF-4	FGF-6	FGF-7
2	POS	POS	NEG	NEG	AREG	bFGF	b-NGF	EGF	EGF R	FGF-4	FGF-6	FGF-7
3	GCSF	GDNF	GM CSF	HB EGF	HGF	IGFBP 1	IGFBP 2	IGFBP 3	IGFBP 4	IGFBP 6	IGF-1	IGF-1 sR
4	GCSF	GDNF	GM CSF	HB EGF	HGF	IGFBP 1	IGFBP 2	IGFBP 3	IGFBP 4	IGFBP 6	IGF-1	IGF-1 sR
5	IGF-2	GM CSF	MCSF R	NT-3	NT-4	PDGFR alpha	PDGFR beta	PDGF AA	PDGF AB	PDGF BB	PLGF	SCF
6	IGF-2	GM CSF	MCSF R	NT-3	NT-4	PDGFR alpha	PDGFR beta	PDGF AA	PDGF AB	PDGF BB	PLGF	SCF
7	SCF R	TGF alpha	TGF beta	TGF beta2	TGF beta3	VEGF	VEGF R2	VEGF R3	VEGF D	BLANK	BLANK	POS
8	SCF R	TGF alpha	TGF beta	TGF beta2	TGF beta3	VEGF	VEGF R2	VEGF R3	VEGF D	BLANK	BLANK	POS

#### f) Metallo proteases (MMP) activity (zymography)

Cell culture supernatants as well as cell lysates prepared as above and clarified by centrifugation (15,000 rpm at 4°C for 15 min; 30μg of protein) were dissolved in non reducing Laemmli buffer and loaded on 8% sodium dodecyl sulfate polyacrylamide gel electrophoresis (SDS-PAGE) co-polymerized with the MMP substrate (20 mg/ml of gelatin, 1% w/v SDS). After electrophoresis, gels were washed twice for 1 hour in 2.5% Triton X-100, incubated overnight at 37°C in incubation buffer (50 mM Tris-HCl, 0.15M NaCl, 10 mM CaCl_2_), fixed, stained in 0.5% Coomassie Blue and destained in 50% methanol– 70% acetic acid. Pictures were taken with a Gel Doc 1000 BioRad. Data were analysed by a computer-based image analysis system (ImageJ, NIH, USA).

#### g) Telomere assay with multiplex-qPCR (MMQPCR)

For the analyses of telomere length variations in the different data set, we used a method based on Cawthon’s, with some modifications [28]. Reactions were carried on by mixing 20 ng of genomic DNA, extracted with Wizard® Genomic DNA Purification Kit (Promega, Milan, Italy), 1x SsoFast EvaGreen Supermix (Bio-Rad), telomere primers (Table 2) in a final volume of 20μl per reaction. An automated CFX96 real-time thermocycler was used (Bio-Rad). The thermal profile and cycling included a first step of 98°C for 2 minutes, for heat-activation of SsoFast EvaGreen Supermix (Bio-Rad) and a second step for anneal and extend the telomeres with 2 cycles of 15 seconds at 94°C and 15 seconds at 49°C. The following steps were made of 40 cycles of 15 s at 94°C, 10 seconds at 62°C, 15 s at 72°C with signal acquisition, 15 s at 82°C with signal acquisition, and a last step melting curve 70.0°C to 95.0°C with increment 0.5°C per second and plate read. The 72°C reads provided the threshold cycle values, determined from semilog amplification plots (Ct) (log increase in fluorescence versus cycle number) for the telomeres, and the 82°C reads measured the Ct values for the amplification of the single β-globin template product while telomere product is fully melted. Results were analyzed with Bio-Rad CFX Manager and exported to Excel (Microsoft) for calculation. The data are expressed as the T/S ratio for each multiplex data set, where T was the measured Ct describing copy number of the telomere template, and S was the copy number of the single copy gene β-globin product used as the internal reference.

### In vitro migration assay

Wound healing assays were performed on cells migrated from spheroids or cultured as monolayers. Cells were plated on 0.1% gelatine (Sigma Aldrich) coated culture plates [11], and once reached confluence were further incubated in serum-free medium for 36 hours. Monolayers were then wounded with a plastic tips as described previously [29], washed with PBS and incubated overnight in the same serum-free medium, with or without the HGF receptor agonist mAb DO-24 [30,31] at different concentrations or with 20% FBS, as positive control. Cells were finally fixed with 3% PFA in PBS and analysed with a Leica DMRB microscope equipped with a digital camera. Migration ability was quantified by calculating the area of wound at time 0 (*t*_*0*_ time of wound) and 24 h after wound (*t*_*14*_). Normalization was obtained by the formula [area(*t*_*0*_)–area (*t*_*24*_)]/area(*t*_*0*_). At least triplicates were quantified for each condition. Data are reported as means ± SD.

### In vivo experiments

A total of 35 C57Bl/6J female mice at the age of six to eight weeks, purchased from Charles River (Research Models and Services, Calco, Italy). The health status of the animals was monitored by a health surveillance program according to the guidelines throughout the experiments. The mice were free of all viral, bacterial, and parasitic pathogens. All animals were housed in groups of three to eight animals. Animals were kept in type 3 clear transparent plastic cages (425 mm x 266 mm x 155 mm) with autoclaved dust-freesawdust bedding and two nestlets^TM^ (each 5 cm x 5 cm) consisting of cotton fibres as nesting material. Additionally, animals were provided with a transparent plastic shelter. They were fed a pelleted and extruded mouse diet ad libitum and had unrestricted access to drinking water. The light/dark cycle in the room consisted of 12/12 h with artificial light. The temperature was 21 +/- 1°C, with a relative humidity of 55+/- 10%, and the air pressure was controlled at 50Pa with 15 complete changes of filtered air per hour (HEPA H 14 filter). The animal room was insulated to prevent electronic and other noise. Mice treated with cyclosporine (25 μg/g) and antibiotics 2 days before the implant and then every second day until 6 days after transplantation. Mice were allocated randomly to one of the four treatment groups, 10 animals injected with spheroids, 10 animals injected with cells in suspension (control), 5 animals were injected with cardiotoxin (CTX; 5 μl of a 10 μM solution; Latoxan, France; Cod:L8102) and 10 animals were injected with cardiotoxin and spheroids, an optimal compromise to obtain significant results and lowering the number of used mice. All the procedures were carried out in double blind. Animals were anesthetized with Avertin (0.2 ml/g) and mechanically ventilated by Minivent (Hugo Sacks, March-Hugstetten, DE). Thoracotomy was performed via the forth left-intercostal space, the parietal pericardium was partially removed and the left ventricle exposed, as described previously [19]. Spheroids (10 for a total cell number of 5x 10^4^ in a volume of 20μl), and the same number of hCPCs in suspension in the same volume, were injected using a 27 gauge-needle in the left ventricle wall. Cardiac damage was induced by injection of CTX in the myocardium apex, as previously described [32]. Cardiospheres were injected near the damaged area shortly after (5 min later). At different time points (1, 3 and 7 days) 3–5 mice for the different treatment group were sacrificed by cervical dislocation, their hearts excised, and perfused with 4% PFA in PBS, 1.41 mM cadmium chloride, embedded in OCT solution (Bio-Optica, Milano, Italy), snap-frozen, and cut in 5-μm sections. Five mice out of 35 (15%) died before the end of the experiment. Tissue sections were stained with Hematoxilin Eosin (HE) or TRITC-phalloidin, mouse anti-human nuclear antigen antibody (see Table 1), followed by FITC-conjugated secondary goat anti-mouse Ig antibodies, rabbit anti-connexin 43 (see Table 1), followed by TRITC-conjugated secondary anti- rabbit Ig antibodies (Alexa Fluor 546) and TO-PRO-3®. Images were taken using a digital scanner Pannoramic Midi 3D HISTEC KFT (Budapest, Hungary) (HE) or a Leica TCS SP2 AOBS spectral confocal scanner microscope (Immunofluorescence). Animal studies were performed according to the guidelines of the Animal Care and Use Committee of the Università del Piemonte Orientale “A. Avogadro” Novara, Italy, following the Decree 116/92 and the D.Ivo #26 of March 4, 2014. The project (n. 17/2013) was approved by the Comitato Etico per la Sperimentazione Animale dell’Università del Piemonte Orientale “A. Avogadro” (C.E.S.A.P.O) on June 22, 2013).

### Statistical analysis

For qPCR and MMQPCR data were expressed as means±SEM from at least three independent experiments, and analysed by unpaired Student’s t test to compare expression between spheroids and cultured cells groups. For wound healing data ANOVA ad Dunnett’s post-test were performed. Statistical computations were performed in GraphPad Prism 4.03 (GraphPad Inc., USA) and values of p≤0.05 or lower were considered statistically significant.

## Results

### Characterization of hCPC spheroids

Spheroids, which were prepared using different number of cells, formed within 20–24 hours, and displayed a relatively homogeneous and number-dependent size distribution (Fig 1A). In view of their use for *in vivo* experiments, in which they were injected through a 27 gauge-needle, most of the experiments were performed with spheroids obtained from 5x10^3^ cells, with a diameter of about 100 μm (Fig 1A). Viability of cells within spheroids was typically 95–98% (Fig 1B), which was maintained, together with their association in aggregates, also after their passage through the 27 gauge-needle (Fig 1A). Spheroids made with more than 5x10^3^ cells disaggregated upon their passage through this needle (Fig 1A).

**Fig 2:**
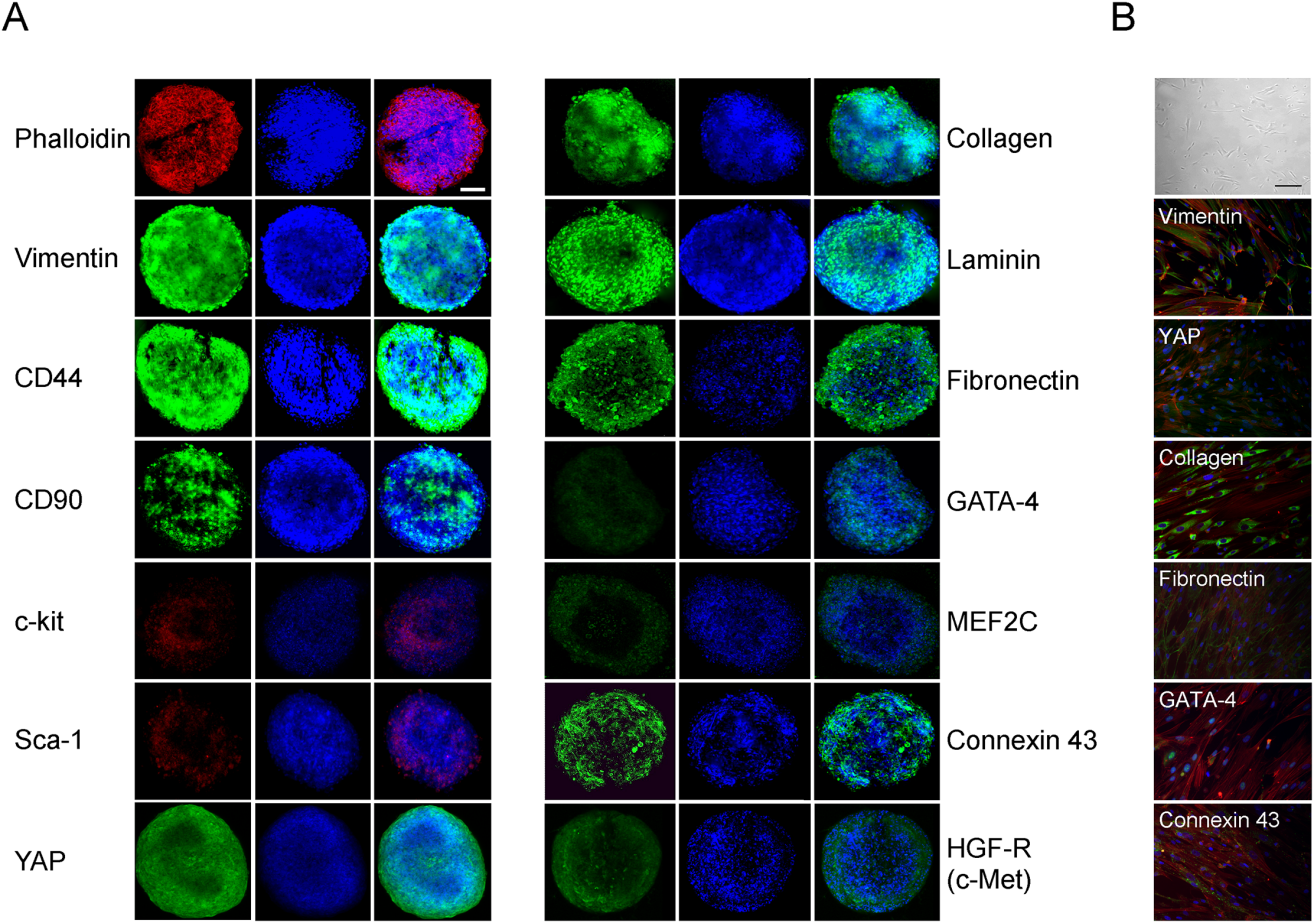
Immunophenotype of hCPC-derived spheroids. (**A**) Confocal microscopy images of spheroids for mesenchymal/stromal, stemness, extracellular matrix, and cardiomyogenic markers, YAP and HGF receptor expression using specific antibodies followed by secondary FITC-labelled antibodies or TRITC-phalloidin. One representative out of the three experiments performed with similar results is shown. Scale bar, 50 μm. (**B**) For comparison, images of hCPCs kept as monolayers at phase contrast microscope (top; scale bar 100 μm) or in immunofluorescence after staining for some of the same markers are shown (20 x magnification).

Cells within spheroids maintained the immunophenotype of the cells from which they were originated [11,19]. Indeed, among others, spheroids expressed the stemness/mesenchymal markers, c-Kit, Sca-1, CD90, CD44 and vimentin and the ECM markers collagen I, fibronectin and laminin (Fig 2A). Moreover, they retained the expression of the cardiac markers connexin 43, GATA-4 and MEF2C, the two latter at lower levels, in agreement with what found in cells in monolayers (Fig 2B, [11,19]). Finally, spheroids expressed also the Met/Hepatocyte Growth Factor Receptor, and the YAP protein, which was recently shown to promote cardiomyocyte proliferation [33]. These same proteins were expressed also in cells cultured as 2D monolayers, as already previously reported [11,19] or shown herein for some markers (Fig 2B).

**Fig 3:**
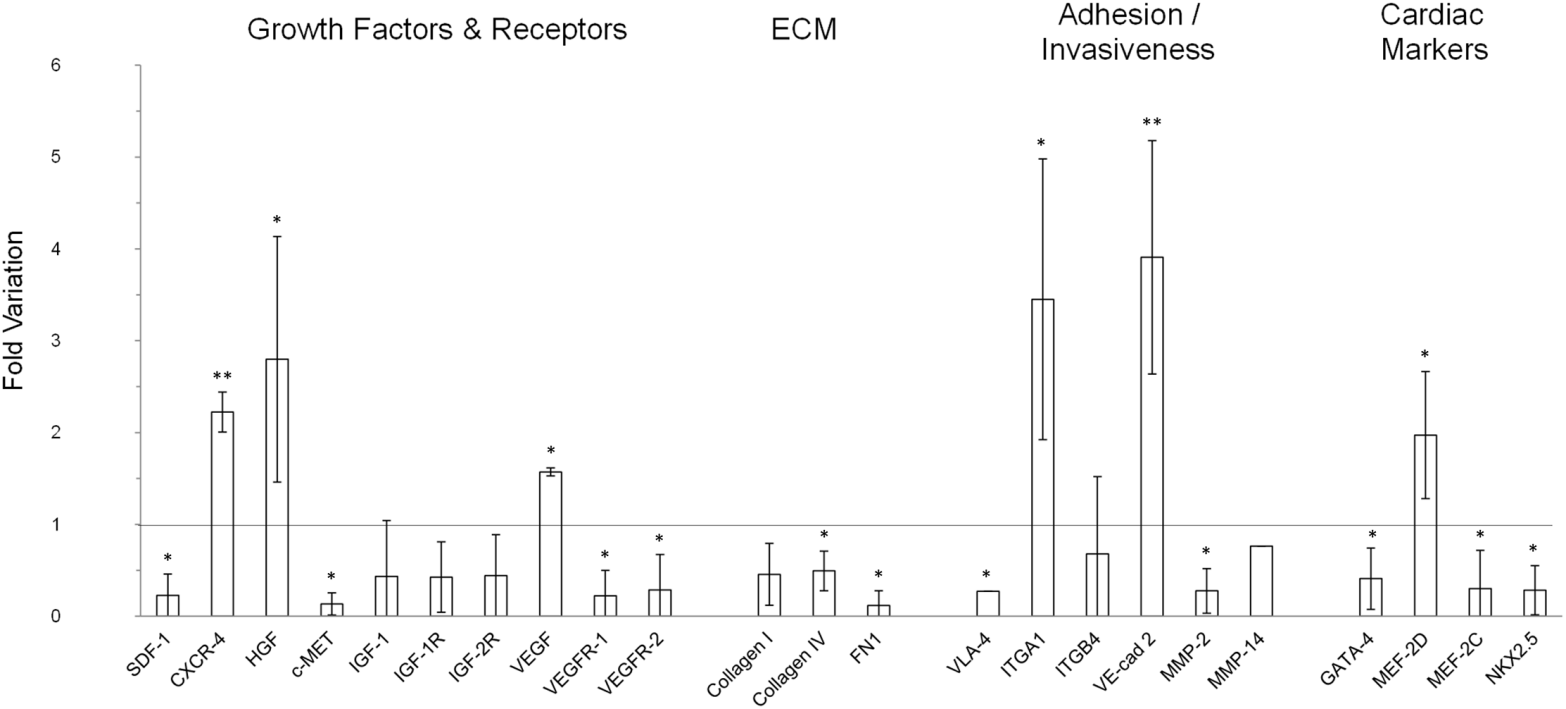
Gene expression of different growth factors, their receptors, ECM molecules, adhesion/invasiveness components and cardiac markers in spheroids and monolayer cultures. Data from qPCR are expressed as fold variation of gene expression in spheroids normalized *vs* monolayers (line). Data are expressed as mean ± SD of at least three experiments. Significant difference of p≤0.05 is indicated as * and p≤0.001 is indicated as **.

**Fig 4:**
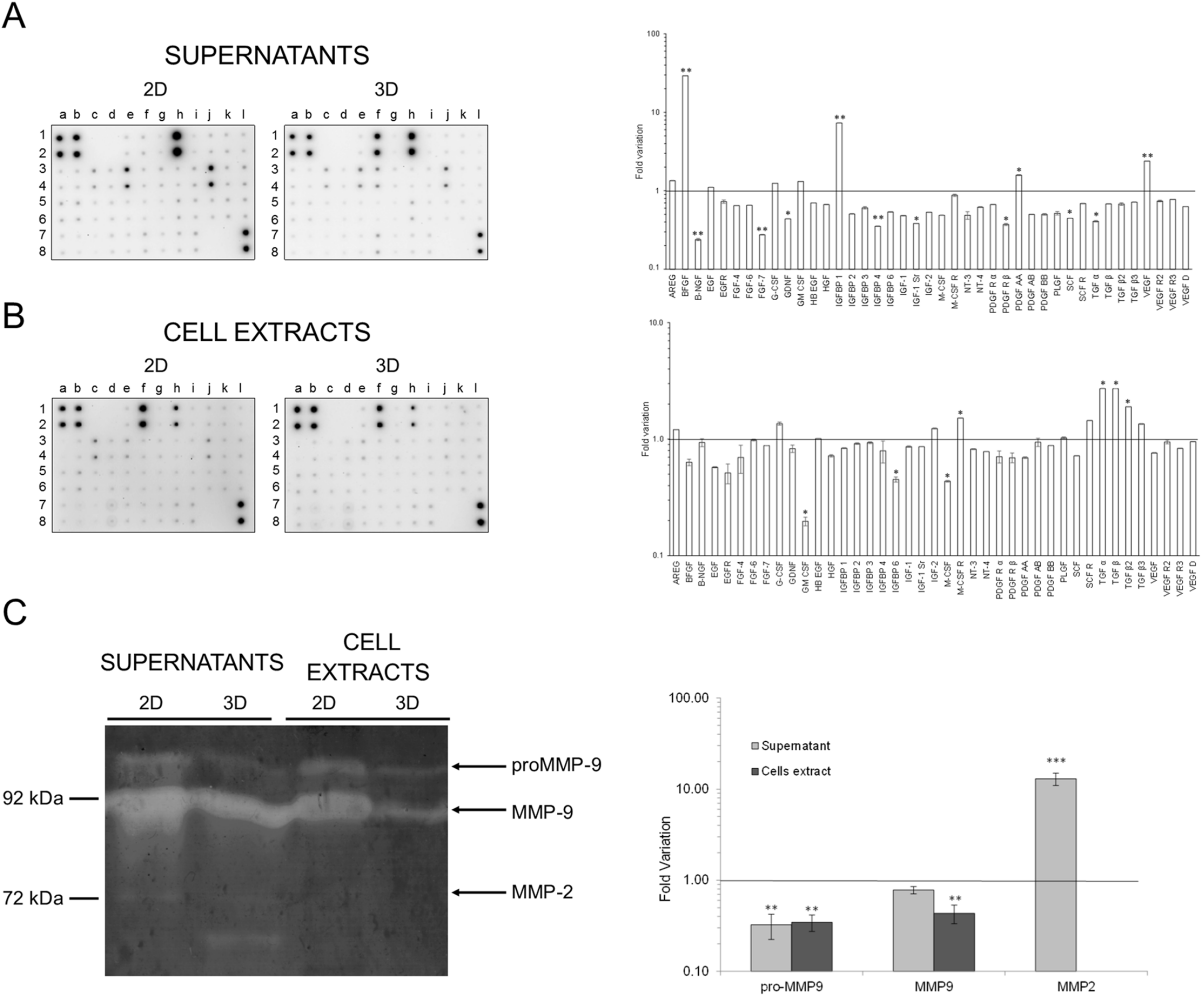
Protein expression of different growth factors, their receptors, and MMP activities. (**A**) Antibody-array analysis of protein expression in supernatants (**A**) from 2D monolayers (left) and 3D spheroids (centre) and in cell extract (**B**) from 2D monolayers (left) and 3D spheroids (centre). Graphs on the right represent fold variation of protein levels in spheroids normalized *vs* monolayers (line). Data are expressed as mean ± SD of at least three experiments. Significant difference of p≤0.05 is indicated as * and p≤0.0001 is indicated as **. (**C**) Activities of MMP-2 and MMP-9 present in culture supernatants and cell extracts from monolayers 2D or 3D spheroids of hCPCs in a Gelatin-substrate zymography. Arrows indicate location of enzymatic activity corresponding to pro- and active forms of MMP-9 and MMP-2. Molecular weight markers (kDa) are shown on the left.

The expression of some of these genes, as well as others belonging to different functional classes, was then analysed by qPCR in hCPCs spheroids and referred to that of hCPCs grown in monolayers (Fig 3). No major differences were found in the genes examined in this assay between spheroids and monolayers. GATA-4, MEF-2C and NKX2.5, which are markers of committed and differentiated cardiomyocytes, were slightly down-regulated in spheroids, where, however, there was an up-regulation of MEF-2D. The same tendency was observed also when we analysed specific growth factors and their cognate receptors. In general these molecules mediate cell homing, proliferation, survival, angiogenesis and motility [34–37]. In spheroids, SDF-1 was down-regulated, while its receptor CXCR4 was up-regulated. HGF and VEGF were up-regulated, while their respective receptors, Met and VEGFR-1 and VEGFR-2 were down-regulated. In the case of the IGF-1 system, both the ligands and the two receptors were down-regulated. The variations were always within 6 folds, and, anyway, also when transcripts were found decreased, the proteins were expressed at levels still detectable in immunofluorescence, as exemplified in the case of the Met protein. Similarly, molecules of the ECM, such as Collagen I, Collagen IV and Fibronectin were down-regulated in spheroids. Also in this case, Fibronectin transcripts were reduced to about 1/4 in spheroids, but still the protein was easily detected in immunofluorescence. Among the receptors for ECM molecules, which should be involved in cell adhesion and migration [38,39], VLA-4 and ITGB4 were down-regulated, while ITGA1 was up-regulated. The VE-cad2 endothelial cadherin, which is essential for vascular development and integrity [40], was up-regulated in spheroids. The expression of different growth factors and their receptors was evaluated also at protein levels both in serum-free supernatants and cell extracts obtained from 2D and 3D cells cultures for 2 days by an antibody array. This assay confirmed a significant expression and release of HGF and VEGF, while IGF-1 was also detectable but at lower levels (Fig 4A). In particular, VEGF expression was higher in the supernatants from spheroids, in accordance with the mRNA data, while HGF expression was lower in comparison to cell monolayers, in contrast to what observed at mRNA analysis. Other growth factors expressed at high levels were EGF, IGFBP-6, and GM-CSF in both 2D and 3D culture supernantants, and bFGF and IGFBP-1 in 3D spheroid supernatants. The latter two factors, as well as VEGF and PDGF-AA were expressed at significantly higher levels in spheroids supernatants. We found that the biologic activity of HGF, as titrated in a scatter assay performed on MDCK, the standard cell line used for this kind of assay [30], in the samples correlated with the quantification obtained at protein levels, i.e, it was 4 times higher in supernatants from 2D cultures, but still detectable in those from 3D spheroids (data not shown). In cell extracts the protein expressed at higher levels was bFGF, followed by EGF, which was present in significant amounts also in cells cultured as monolayers (Fig 4B). All the other proteins were detectable at similar levels in the two conditions of cell growth, e.i. as monolayers and as spheroids.

Another biological activity associated to stem cells and their migration potential particularly important in regenerative responses of injured organs [36,37] is their invasiveness, which is supported by lytic enzymes specific for the ECM components, such as MMPs [41]. qPCR analysis showed that in spheroids MMP2 was down-regulated, while the level of MMP14 transcripts did not change (Fig 3). The lytic activities in the cell culture supernatants and in the cell extracts were directly tested in a zymograph assay on gelatin (Fig 4C). Besides activated MMP2, which was mainly detectable in spheroid supernatants, MMP9 and its precursor were present at high levels in all samples, although in higher amounts in samples derived from 2D cultures, both supernatant and cells. As in the case of growth factors and their receptors, the fact that no perfect correlation was found between the mRNA and the protein analysis can be partially explained by considering that mRNA was extracted after 1 day culture, while proteins were analysed after 2 days of cell culture, to allow their secretion at detectable levels. All together these data indicate that upon their spatial organization in 3D spheroids, hCPCs maintain easily detectable levels of MMPs, with a differential expression of the different isoforms. This implies that these cells have not undergone important changes relative to their previous culture conditions as 2D monolayers for what is their invasive potential.

**Fig 5:**
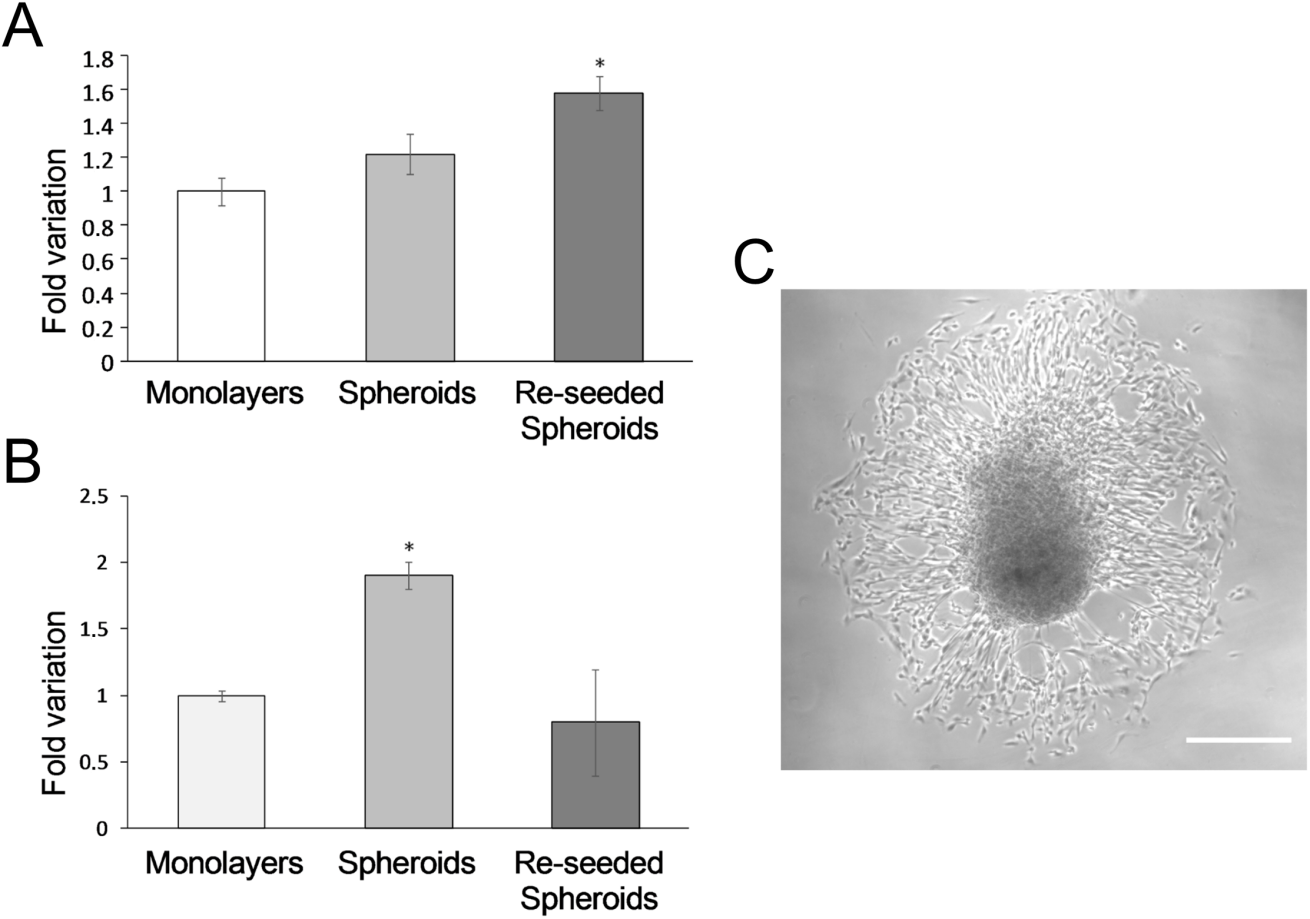
Telomere length (A) and telomerase expression (B). Variation in spheroids and reseeded spheroids vs monolayers. Data expressed as mean ± SD. (**C**) Cell spreading from spheroids after reseeding on culture 2D plates. Scale bar 120 μm.

The telomerase/telomere system provides an index of the cell growth potential and has also been applied to hCPCs and their committed progeny [42,43]. Cells within spheroids were found to display longer telomeres in comparison to cells in monolayers and a more significant elongation was observed in the cells derived from them after seeding and growth as monolayers (p<0.001) (Fig 5A and 5C). The telomerase activity, which was measured in parallel, was found to be transiently increased in 3D spheroids, but then reduced at basal levels in cells growing as monolayers after reseeding the spheroids (Fig 5B). These data indicate that hCPCs retain the typical stem cell ability of activating/deactivating telomerase [42].

### In vitro migration of hCPCs from spheroids

**Fig 6:**
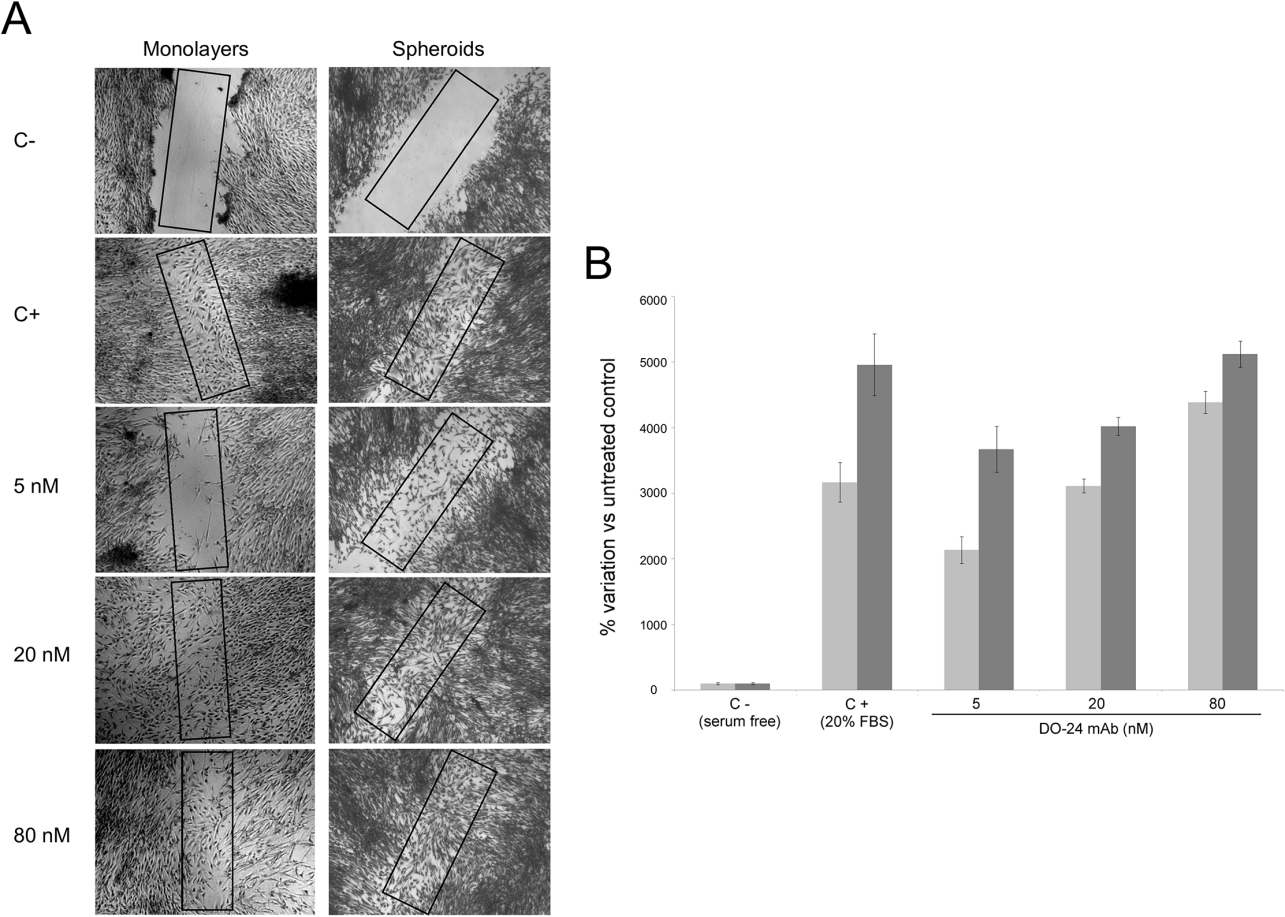
Migratory activity of hCPCs kept as monolayers or of cells migrated from seeded spheroids (wound healing assay). (**A**) Quiescent cell monolayers were “wounded” with a pipette tip and incubated for 24 hours in the absence (C-) or presence of 20% FBS (C+) or of different concentrations of the HGF receptor agonist mAb DO-24 (5, 20, 80 nM respectively). (**B**) Quantitative analysis calculated as % variation of cell numbers in the rectangles depicted in Fig 5A relative to untreated samples. The assay was repeated three times with triplicates and one representative assay is reported. Images were taken at 5x magnification. After 24 hours healing was significantly induced by 20% FBS and mAb DO-24 treatments. Cells derived from spheroids (dark grey bars) were more efficient than cells from 2D cultures at any condition (light grey bars).

Since spheroids were projected for *in vivo* transplantation experiments, in which their cells were expected to dissociate, migrate and eventually engraft, a wound healing assay was performed to assess the *in vitro* migratory potential of the cells derived from them. The assay was performed on confluent monolayers obtained from cells migrated from cultured spheroids. As control reference, hCPCs were cultured in standard conditions. The assay also included an agonist of the Met/HGF receptor [30] in order to evaluate the response to a possible pharmacological modulation. Cells in both culture types migrated in response to 20% FBS. However, the healing rate in the case of cells migrated from spheroid was higher, since wound healed in 16 hours (data not shown), while in the case of 2D cultures 24 hours were necessary for complete healing (Fig 6A). The same trend was observed also upon Met/HGF receptor stimulation, where a clear dose response migration of the cells at 24 hours is reported (Fig 6B).

### hCPCs within spheroids engraft in the heart walls of C57/B6 mice

**Fig 7:**
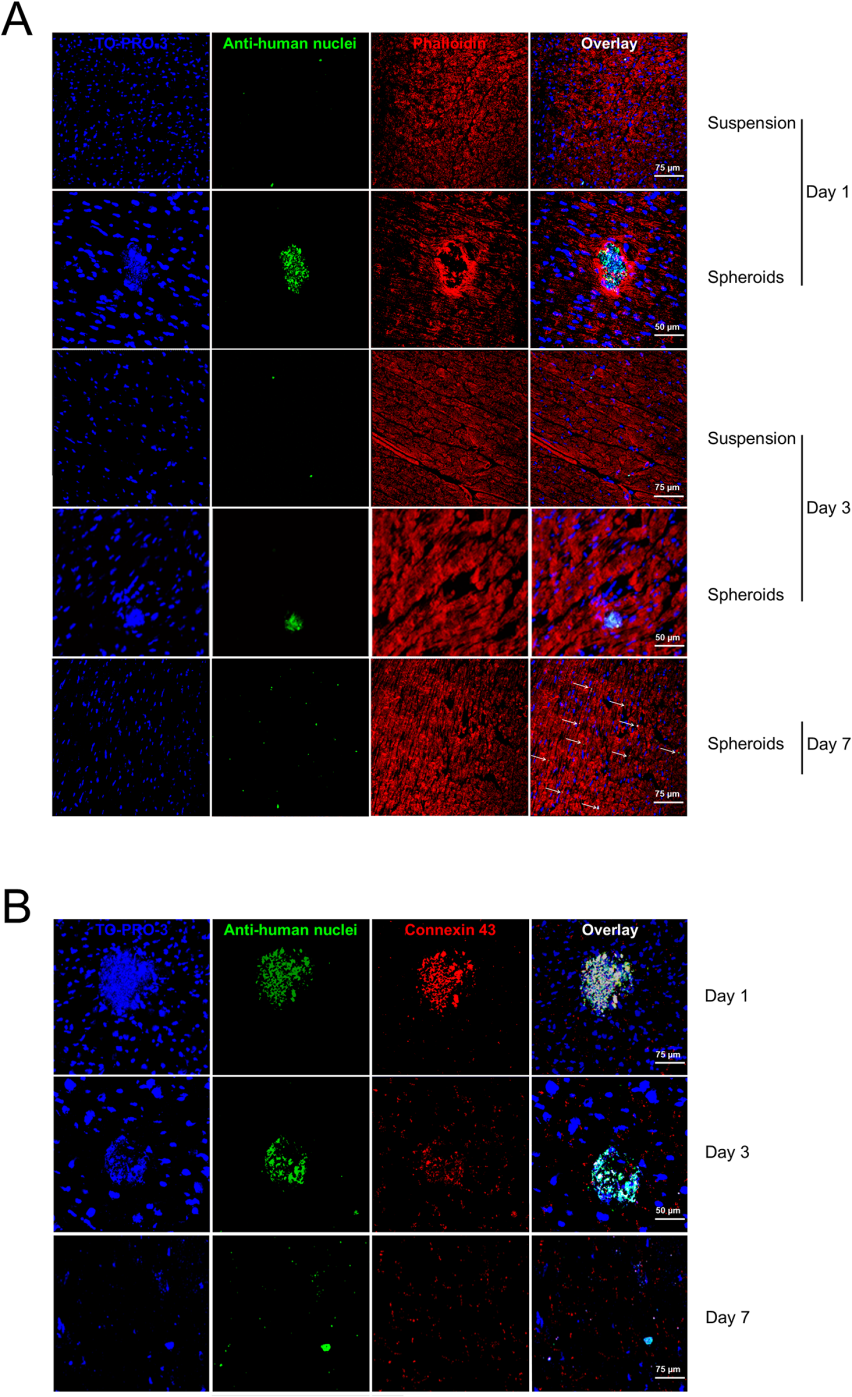
*In vivo* transplantation of spheroids in healthy mice. (**A**) Myocardium sections from mice after transplantation with hCPCs in suspension or as spheroids, at different time points, were stained with TO-PRO3 (blue) to show all nuclei, anti-human nuclei antibody (green), and phalloidin (red) for actin and analysed by confocal microscopy. (**B**) Myocardium sections from mice transplanted with spheroids were also stained at different time points with TO-PRO3 (blue), anti-human nuclei antibody (green), and anti-connexin 43 (red). Representative experiments out of three performed are shown.

To evaluate the possibility that spheroids could be used as a fast delivery system for hCPCs *in vivo*, they were injected into the left ventricular wall of immunosuppressed mice. Two sequential experiments were run. In the first set healthy animals were injected with the same number of cells either in suspension upon release with trypsin and EDTA from monolayers or in spheroids, and then sacrificed at different time points. At day 1 after transplantation, cell spheroids were still clearly detectable, as shown by their staining with anti-human nuclei antibodies (green nuclei, Fig 7A and 7B) and connexin 43 (red, Fig 7B). On the contrary, the administration of the same amount of hCPCs as cell suspension did not allow a consistent inclusion of human cells into the myocardium. At day 3 a number of cells migrated from spheroids, as demonstrated by the loss of the compact spherical morphology and the presence of dispersed cells into the myocardium matrix (Fig 7A and 7B). At the same time point, rare and sparse hCPCs were detectable when administered as cell suspension. At day 7, hCPCs derived from spheroids were still detectable, while no cells were detectable in the case of cell suspension injection. At this time point cells were fully dispersed within the myocardium wall (Fig 7A and 7B).

**Fig 8:**
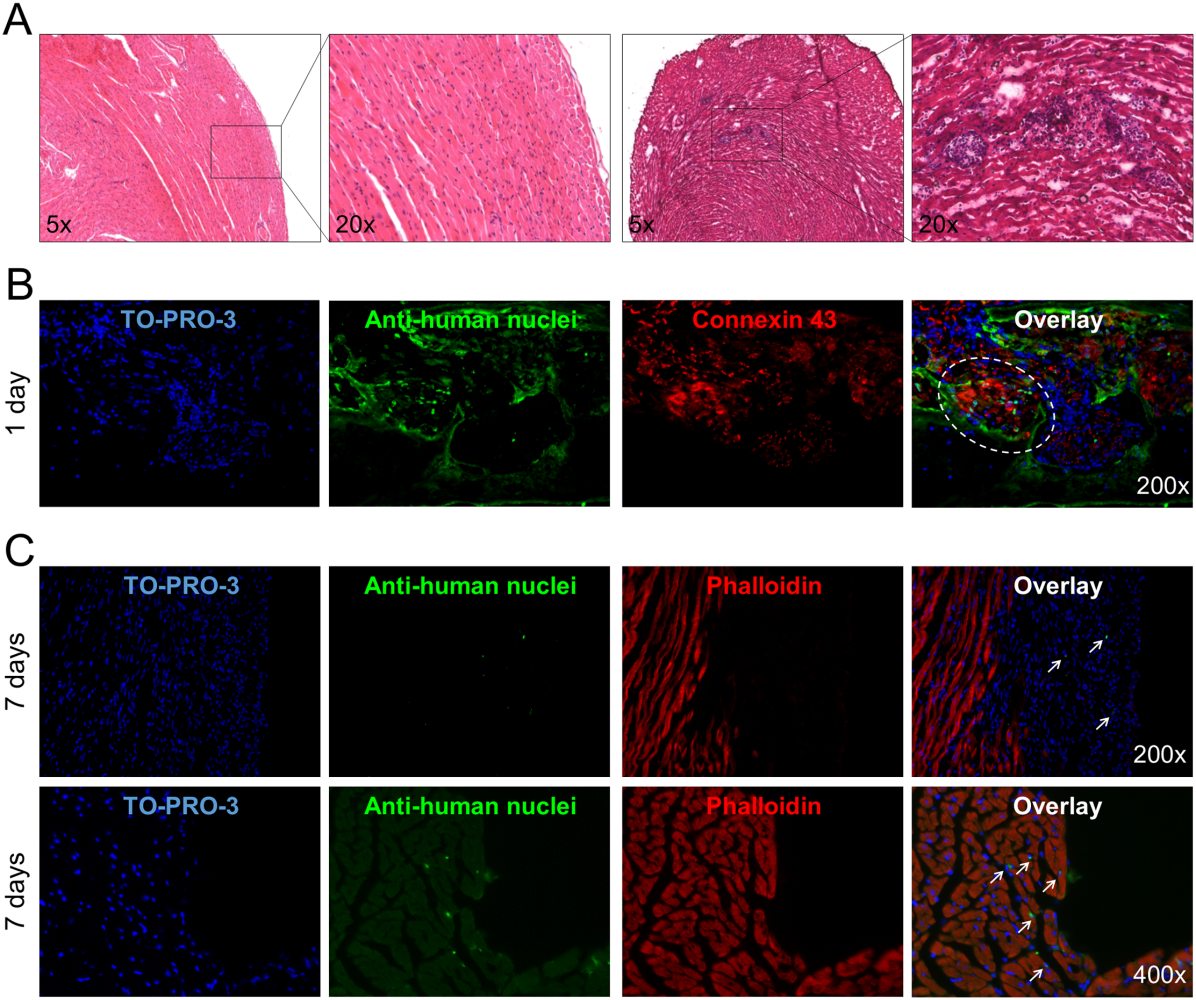
*In vivo* transplantation of spheroids in myocardium-injured mice. (**A**) Evaluation of cardiotoxin-induced injury in the myocardium wall at two magnifications (right) compared to healthy myocardium (left) in Hematoxylin-Eosin staining. (**B**) Myocardium sections from myocardium injured mice transplanted with spheroids were stained with TO-PRO3 (blue) to show all nuclei, anti-human nuclei antibody (green), and connexin-43 or phalloidin (red) for actin and analysed by confocal microscopy. Representative experiments out of three performed are shown. The circle and arrows show the engrafted spheroid 1 day after the injection and the dispersed hCPCs 7 days after the injection, respectively.

The second set of experiments was aimed to assess if cells could engraft and survive in the hostile environment induced by CTX injury, which mimics the damage induced by myocardial infarction [32]. Myocardium injury was confirmed by hematoxilin-eosin staining (Fig 8A). Also in this case spheroids were detectable one day after the injection (Fig 8B) and after one week few cells were still present in the area of injury (Fig 8C). In both cases the injured site of the myocardium was deeply infiltrated with mononuclear inflammatory cells. It can thus be concluded that the cells can engraft in the injured myocardium.

## Discussion

Stem cell therapy has always been envisaged as the possibility to repair injured heart by replacing the dead cells [2,44]. The major obstacle to this approach is that most of the transplanted cells, independently of their origin and histotype, are lost in a short time (>99% within 24h) when administered as cell suspension [14]. This drawback could be partially overcome if cells are administered in the form of aggregates, such as cardiospheres [6,12,45], cell sheets [20] or sheet fragments [21]. The 2D/3D structures likely reproduce, at least in part, the natural environment to which cells are accustomed, comprising a supporting ECM [46] In this work we show that it is possible to produce spheroids of human CPCs with an easy, quick and cheap approach and that cells within these spheroids maintain the markers of the original progenitor cells, while enhancing some biological characteristics, such as proliferation and migration potentials. Moreover, we show that this approach is suitable for producing spheroids that can be transplanted in the heart wall, since, when transplanted in healthy or myocardium injured mice, spheroids could release cells able to engraft and to remain detectable for at least 1 week.

Spheroids were generated using the temperature sensitive methylcellulose substrate and were obtained with little manipulation (only one step) and in shorter time (less than 24h) in comparison to other protocols used to prepare cell aggregates, such as cell sheets [20] and cardiospheres [12]. The short time of culture necessary to obtain the spheroids allowed to maintain the expression of early commitment cardiac markers (c-kit, Sca-1, GATA-4, MEF2C, MEF2D and connexin 43) as well as of the stemness/mesenchymal markers (CD90, CD44 and vimentin). Furthermore, we demonstrate that cells into spheroids maintained the expression and the release of many growth factors and the expression of their cognate receptors, which are generally involved in cell homing, proliferation, survival, angiogenesis and motility [36].

Interestingly, spheroids show some differences in the levels of expression of some of these factors compared to 2D monolayers, with the general tendency of their down-regulation. For example, for SDF-1/CXCR4 axis, which mediates mainly cell homing to injured organ [47,48], the ligand was down-regulated, but the receptor was up-regulated. In the case of IGF-1 and its receptors, mostly involved in cells survival, proliferation and differentiation [49], the three molecules were down regulated. HGF and VEGF, which are involved mainly in morphogenesis/tubulogenesis and vasculogenesis [50], were both upregulated, while their receptors were down-regulated. The analysis of protein expression by the antibody array included also other factors and revealed differential release of few factors. The more evident difference was that spheroids released huge amounts of bFGF and significant amounts of IGFBP-1, the other growth factors being generally released within the same range. All together, these data can suggest a higher CXCR4- and bFGF-mediated homing potential of cells within spheroids, and an increased potential to promote VEGF-mediated angiogenic responses in their microenvironment. Indeed bFGF has been reported to enhance engraftment of mesenchymal stem cells for cardiac repair better than VEGF and IGF-1 [25] by protecting cells from the post-ischemic microenvironment [51]. Concordantly, the main proteins expressed by cells, both in 2D and in 3D, were bFGF and IGFBP-1. The lower expression of IGF in supernatants of spheroids could be compensated by the higher expression and release of IGFBP-1, which can stabilize the short-lived circulating IGF [52]. Also the VE-cad2 endothelial marker was shown to be up-regulated in spheroids. Spheroids contained highly viable cells, likely because their size around 100 μm is largely within the limits of oxygen diffusion in compact highly cellularized tissues and the short time needed for their assembly. The fact that cells in spheroids show some neovascularization potential is noteworthy and could predispose for the oxygenation requirement of cells in solid compacted structures after the engraftment [53]. The decreased expression of Met, which anyway was still detectable in immunofluorescence, did not affect the capability of the cells migrated from spheroids to respond to HGF in a motility assay, such as wound healing.

Cells in spheroids displayed a higher replicative potential relative to cells in monolayers, since they showed an increased length of telomeres. An even more significant elongation was observed in the cells derived from spheroids, which were reseeded and grown as monolayers. The increased telomere length was supported by an increased level in the expression of telomerase, which provided an index of the growth potential of hCPCs and of their committed progeny. Strong activation of telomerase has been detected in most human tumour tissues, while normal somatic cells lack this activity. Germinal and stem cells are able to activate at low levels or to down-regulate this enzyme, depending on the replicative needs of the cells [54]. The observed increased activity of telomerase in spheroids in respect to monolayers may indicate that the spheroid condition predispose cells to replication. Furthermore, cells derived from spheroids after seeding on culture plates were able to down-regulate telomerase at a level typical of the cells grown as monolayers. Thus after telomerase reactivation, the cells that originated from spheroids down-regulated it to basal levels since a sufficient telomere length was achieved to sustain proliferation [55]. These data suggest that the culture condition in spheroids has the effect of rejuvenating hCPCs, which acquire the potential of more cell cycles [56]. This observation is in line with what recently reported for cells from ciliary body, which, if in spheroids, could be reprogrammed in the presence of only Oct4, not requiring Klf4, which otherwise was necessary if cells were maintained as monolayers [57]. The fact that hCPCs are able to modulate positively and negatively telomerase depending on the culture conditions is noteworthy, since it indicates that the cells have not a tumoral behaviour but retain a stem cell profile. The proliferative potential of cells within spheroids is also in line with their expression of YAP, a transcriptional cofactor involved in Hippo signalling pathway [58]. YAP promotes proliferation of embryonic cardiomyocytes by activating the insulin-like growth factor and Wnt signalling pathways and acting as dynamic sensor of substrate mechanics. Moreover, its constitutively activated form can stimulate cardiac regeneration in the adult heart and can improve contractility after myocardial infarction in mice [33]. These data, apparently contradictory with the observed down-regulation of the IGF-1 axis, can be reconciled by supposing that cells in spheroids acquire a more stemness phenotype, i.e. they are more quiescent, but have an enhanced proliferation potential that they will display once spheroids are reseeded in 2D cultures. Similar conclusions were reached when cardiospheres, which are spontaneously generated by culturing in suspension clones of cells derived from heart biopsies, and cells dissociated from them and cultured as monolayers, were compared [45].

Cells within spheroids displayed also good tissue adhesive properties: indeed they synthetize ECM components (collagen I, collagen IV, laminin, fibronectin), and in the meantime express integrins at their surfaces. Also in this case, cells in spheroids displayed the tendency to lower the expression of most of these molecules; however, they were clearly detectable in immunofluorescence. These molecules are important not only for adhesion and migration; they can also impart signalling for cell survival, proliferation and differentiation [38,59] which are transduced within cells through integrin receptors [39,40]. In these pleiotropic cell responses, signals from ECM and growth factors act in dynamic cooperation, both in vitro and in vivo [60]. Cells in spheroids displayed also a superior propensity for migration than cells grown as monolayers, as shown in the wound healing assay. We also assayed the effects of a possible pharmacological modulation of this process by using an HGF receptor agonist. HGF was chosen as a prototype growth factor, since it is a recognized motogenic factor for mesenchymal stem cells of different origin and species *in vitro* [61], *ex vivo* [48] and *in vivo* [49], and in particular was applied in the field of myocardium. Moreover, compared to SDF-1, a different homing factor, HGF has already shown to promote cardiac commitment [49,61] anti-apoptotic activity on cardiomyoblasts [31] and *in vivo* cardioprotection [62]. On the other side HGF is only one of the many growth factors and cytokines released from injured organs to recruit cells for their regeneration and repair [36]. Finally, also the expression, release and activity of matrix metalloproteases (MMPs)-molecules known to play a role in MSC migration [63]- suggested that spheroids had acquired a more favourable migratory and engraftment phenotype compared to monolayer cultures. In details MMP-9 lytic activity was lower in both supernatants and cell extracts from spheroids, while MMP-2 activity was secreted only by spheroids. Down-regulation of MMP-9 in myogenic cells enhances their engraftment in dystrophic muscle [64], and thus its slight decrease in spheroids could explain their better engraftment ability. By contrast, an increase of MMP-2 was associated with a better migration, proliferation and engraftment of low adherent skeletal myoblasts [65]. Thus, the spheroid phenotype, while maintaining all the properties of the cells cultured as monolayers, seems to be more appropriate for the *in vivo* administration than their counterpart derived from 2D cultures.

We performed *in vivo* experiments to support these hypotheses and the data, although preliminary, confirmed these expectations. hCPCs derived from spheroids remained detectable in the myocardium of healthy C57/B6 mice for at least 1 week after transplantation. Furthermore they were able to migrate from spheroids and to disperse in the cardiac matrix, while cells injected as suspensions were barely detectable 1 day after transplantation. These data are in line with those reported by Li et al., 2010, who compared the in vivo fate of cardiospheres vs cells dissociated from them [45]. These observations are probably due to the fact that spheroids can entrap into the myocardial interstices and can facilitate ECM-mediated engraftment. We planned short time point experiments since the goals of these preliminary *in vivo* data were to confirm the potential of this rapid technique and to show the impact of this procedure on cell phenotype. Due to this short time of observation cells are not expected to undergo significant differentiation. Furthermore, we have chosen a low number of cells to be transplanted as spheroids in contrast to similar experiments performed with cell aggregates, i.e. cell sheets [19,20] or cardiospheres [12,66], in order to reproduce a more realistic scenario for hCPC transplantation. Future long term experiments focusing on the recovery of the cardiac function in specific *in vivo* models will definitively elucidate the real potential of this procedure, although our preliminary data herein show that the spheroids prepared with this methodology are able to engraft also in a model of myocardial infarct.

Besides the advantage linked to their physical status of cell aggregates shown by the *in vivo* experiments described above, spheroids has the additional advantage to better preserve stem cell properties, i.e. self-renewal through stable telomere length and migration ability triggered by growth factors and cytokines. These traits, which are typical of embryonic stem cells during development, are essential for the regeneration and healing of injured organs in adults. Indeed a phase 2 trial with cardiospheres, similarly shaped multicellular structures, are already under way [6]. Herein we show that ready-to-implant scaffold-less aggregates of human cardiac progenitor cells can be produced with an easy, economic and fast protocol, thus making spheroids good candidates for future cell therapies applied to myocardium, drug discovery and screening.
