# Correction: Human Cardiac Progenitor Spheroids Exhibit Enhanced Engraftment Potential

**DOI:** 10.1371/journal.pone.0141632

**Published:** 2015-10-23

**Authors:** Francesca Oltolina, Andrea Zamperone, Donato Colangelo, Luca Gregoletto, Simone Reano, Stefano Pietronave, Simone Merlin, Maria Talmon, Eugenio Novelli, Marco Diena, Carmine Nicoletti, Antonio Musarò, Nicoletta Filigheddu, Antonia Follenzi, Maria Prat

The fourteenth and fifteenth authors, Antonia Follenzi and Maria Prat, should be noted as contributing equally to this work.

In the Author Contributions section, Antonia Follenzi (AF) should be listed as one of the persons who conceived and designed the experiments, contributed reagents/materials/analysis tools, and wrote the paper in addition to analyzed the data.
